# Amino Acid and Acylcarnitine Levels in Chronic Patients with Schizophrenia: A Preliminary Study

**DOI:** 10.3390/metabo11010034

**Published:** 2021-01-05

**Authors:** Irina A. Mednova, Alexander A. Chernonosov, Marat F. Kasakin, Elena G. Kornetova, Arkadiy V. Semke, Nikolay A. Bokhan, Vladimir V. Koval, Svetlana A. Ivanova

**Affiliations:** 1 Mental Health Research Institute, Tomsk National Research Medical Center of the Russian Academy of Sciences, Aleutskaya Str., 4, 634014 Tomsk, Russia; kornetova@sibmail.com (E.G.K.); semkeniipz@tnimc.ru (A.V.S.); bna909@gmail.com (N.A.B.); ivanovaniipz@gmail.com (S.A.I.); 2 Institute of Chemical Biology and Fundamental Medicine, Siberian Branch of the Russian Academy of Sciences, Lavrentyev Avenue 8, 630090 Novosibirsk, Russia; alexander.chernonosov@niboch.nsc.ru (A.A.C.); kassakinm@gmail.com (M.F.K.); koval@niboch.nsc.ru (V.V.K.); 3 University Hospital, Siberian State Medical University, Moskovsky Trakt, 2, 634050 Tomsk, Russia; 4 Department of Psychiatry, Addictology and Psychotherapy, Siberian State Medical University, Moskovsky Trakt, 2, 634050 Tomsk, Russia

**Keywords:** amino acid, acylcarnitine, schizophrenia, biomarker, metabolomics

## Abstract

Amino acids and acylcarnitines play an important role as substrates and intermediate products in most of pathways involved in schizophrenia development such as mitochondrial dysfunction, inflammation, lipid oxidation, DNA damage, oxidative stress, and apoptosis. It seems relevant to use an integrated approach with ‘omics’ technology to study their contribution. The aim of our study was to investigate serum amino acid and acylcarnitine levels in antipsychotics-treated patients with chronic schizophrenia compared with healthy donors. We measured serum levels of 15 amino acids and 30 acylcarnitines in 37 patients with schizophrenia and 36 healthy donors by means of tandem mass spectrometry. In summary, patients with chronic schizophrenia had an altered concentration of a few amino acids and acylcarnitines in comparison to the healthy probands. Further research is needed to assess and understand the identified changes.

## 1. Introduction

Schizophrenia is a heterogeneous, severe mental illness that affects 0.5–1% of the population worldwide [1]. Schizophrenia is often associated with stigma, with about half of patients experiencing stigmatizing social attitudes that impair patient’s quality of life [2]. To understand the pathogenesis of schizophrenia, abundant factors and mechanisms have been considered and discussed e.g., mitochondrial dysfunction, low-grade inflammation, DNA damage, oxidative stress, and apoptosis [3,4,5,6]. These abnormalities of the metabolic pathways in patients with schizophrenia may well be reflected in their metabolomic profiles [7]. Amino acids and acylcarnitines play an important role as substrates and intermediate products in the most of these pathways [8,9]. The first reference of the amino acid profile change in schizophrenia was in 1980 when J. Kim et al. showed a decrease in glutamate concentration in the cerebrospinal fluid of patients [10]. After that, most research focused on glutamate and gamma-aminobutyric acid, but recently other metabolites were also intensively studied [11,12,13,14]. Y. He et al. showed aberrations in the glutamine and arginine metabolic pathways and associated genetic risk factors, which according to them suggest a relationship with memory deficits associated with schizophrenia [15]. After addition of amino acid supplements, such as l-lysine [12], glycine [16], d-serine [17], d-alanine [18], or fatty acids [19] to the diet of patients with schizophrenia, symptoms of disease significantly improved. Recently, acylcarnitines were regarded as markers of fatty acid and amino acid oxidation disturbance [20]. There is a great deal of evidence in the literature about multifactorial roles for these compounds in neuroprotection. For instance, acylcarnitines can improve the overall energy status of the brain and alter the biosynthesis patterns of some neurotransmitters providing high-energy acetyl groups. They also can be involved in modulating proteins and gene expression and improve mitochondrial function through improvements in membrane lipid content and enzyme activities [8]. Little is known about changes in acylcarnitine levels in schizophrenia. Analysis of the acylcarnitines showed increase levels of C4-OH (C3-DC) and C16:1, and decrease of C3, C8, C10, C10:1, C10:2, C12, C14:1-OH, C14:2, and C14:2-OH in the blood plasma of patients with schizophrenia compared with healthy individuals matched for age and sex [14]. K. Kriisa et al. demonstrated a rise in the level of a few acylcarnitines in drug-naïve patients with schizophrenia [21]. 

Taking into account the literature data, we hypothesize that in schizophrenia the profile of amino acids and acylcarnitines will be altered.

Thus, the aim of our study was to investigate serum amino acid and acylcarnitine levels in schizophrenia patients compared with healthy donors. 

## 2. Results

The study included 37 patients with schizophrenia and 36 healthy individuals (Table 1). All patients received antipsychotic treatment in antirelapse and maintenance dosages before admission to the clinics. However, they did not take medicine regularly and were admitted to the clinics with an acute condition. 

We found a significant decrease in the concentration of some amino acids: valine (*p* < 0.001), aspartate (*p* < 0.001), citrulline (*p* < 0.001), glycine (*p* = 0.003), arginine (*p* = 0.021), and ornithine (*p* = 0.002) (Table 2) in patients with schizophrenia in comparison to healthy subjects.

The analysis of acylcarnitine profile established a significantly decreased level of long-chain species: C14 (*p* = 0.019), C14-OH (*p* = 0.018), C16-OH (*p* < 0.001), C16:1 (*p* = 0.003), C16:1-OH (*p* < 0.001), C18 (*p* < 0.001), C18-OH (*p* < 0.001), C18:1 (*p* = 0.029), C18:1-OH (*p* < 0.001), C18:2-OH (*p* = 0.003), and short-chain acylcarnitine C5:1 (*p* < 0.001) and increased C4-DC (*p* < 0.001) in patients with schizophrenia in comparison to the controls (Table 3).

**Figure 1:**
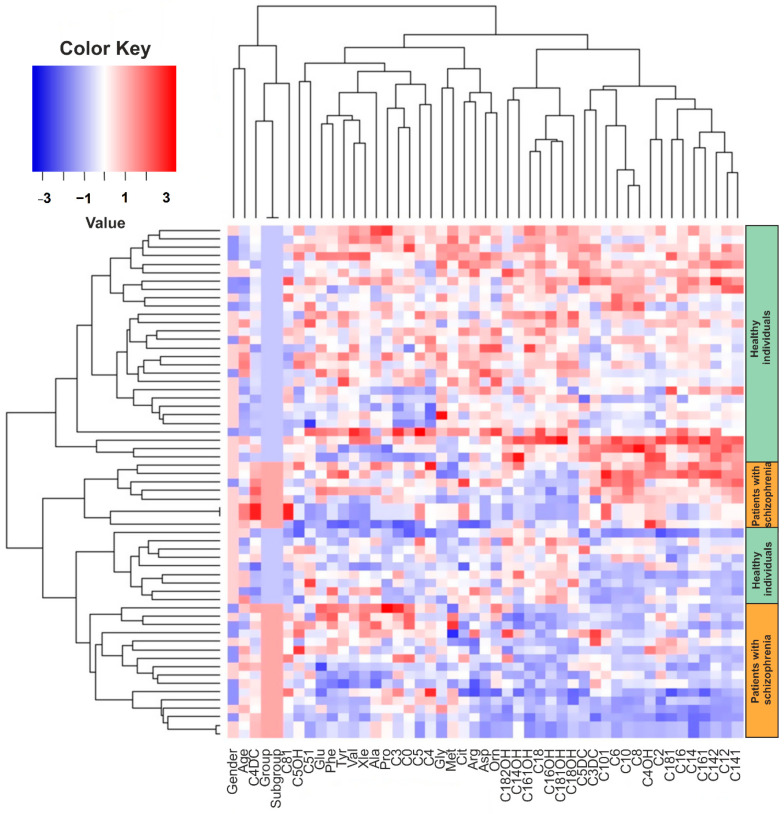
Hierarchical analysis of the amino acids and acylcarnitine changes dataset. The rows represent the patients′ samples and the columns represent the analyzed parameters (amino acids, acylcarnitine, groups, age). In the heatmap, the color codes a dimensional scale, where the blue gradient corresponds to the decrease of values and red gradient corresponds to increase of values in the patient group in comparison to healthy control group.

In the next step, we analyzed metabolic data using multivariate analysis methods. First, we clustered the data using the hierarchical cluster analysis method (Figure 1). Data were prefiltered to outliers for each metabolite. The criterion of values from the total distribution as outliers was set the 1.5-time intraquartile range (1.5 × IQR). Thus, if the value is 1.5 times lower or higher than the intraquartile range, it is considered to be an outlier and is not taken into account in the calculations.

According to cluster analysis, among the acylcarnitines of long chain fatty acids, a group of metabolites was isolated: C18, C18-OH, C18:1-OH, C18:2-OH carnitines (stearic FA derivatives), C16-OH, C16:1-OH carnitines (palmitic FA derivatives), C14-OH carnitine (derivative myristic FA), whose concentrations were reduced in the group of patients compared with the control group. At the same time, C4-DC showed an inverse relationship—in the group of patients with schizophrenia, its concentration was higher. These data correlate with univariate analysis data (Table 2), where a decrease in the concentration of long-chain acylcarnitines (C14–C18) and an increase in the concentration of C4-DC were also found in the group of patients with schizophrenia.

**Figure 2:**
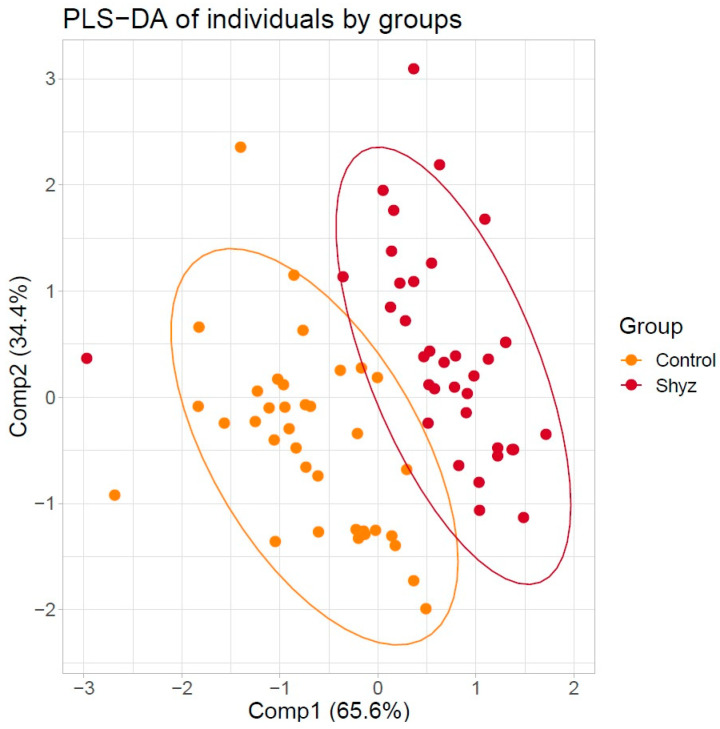
Scatter plot of 73 individuals by the first two principal components extracted from amino acids and acylcarnitines concentrations by partial least squares discriminant analysis (PSL-DA). Two distinct clusters were noted. Clusters are marked using red color for group patients with schizophrenia and orange color for healthy individuals.

Partial least squares discriminant analysis (PLS-DA), one of the most commonly used in metabolic studies, was used to classify samples into groups. The result of dividing the samples into groups is shown in Figure 2.

## 3. Discussion

In our study, we for the first time determined the amino acid and acylcarnitine levels in patients with schizophrenia within a Russian population using metabolomic techniques. We found that patients with schizophrenia had a decreased concentration of valine, aspartate, citrulline, glycine, arginine, and ornithine as well as C14, C14OH, C16OH, C16:1, C16:1OH, C18, C18OH, C18:1, C18:1OH, C18:2OH, and C5:1 and increased concentration of C4DC in comparison to the healthy probands.

Schizophrenia is a socially significant disease due to its high prevalence, progression, severity of social consequences, and high economic cost burden. Progress in the study of the neuropathology of schizophrenia can be associated with genetic, proteomic, metabolomic research, and the search for biomarkers of endogenous mental disorders. Gray matter decreases in the anterior cingulate have been reported as markers of genetic liability to psychosis, while reductions in the superior temporal gyrus and cerebellum may be the first onset of the disease markers [22]. Quantitative proteomic analysis using mass spectrometry identified 27 proteins specific to schizophrenia [23].

During the last decade, several authors have reported alterations of amino acid metabolism in schizophrenia, but most of this data was obtained in small cohorts of patients and it contained ambiguous and inconsistent results. This may be due to the different duration of the disease, the treatment, and other factors. In our study, we discovered a significant decrease in the concentration of valine, aspartate, citrulline, glycine, arginine, and ornithine in schizophrenia.

It is known that arginine metabolism is altered in schizophrenia, particularly indicated by reduced expression of genes involved in the regulation of L-ornithine and polyamine metabolism, decreased plasma arginase activity, and a positive correlation between the serum levels of L-ornithine and the duration of illness [24]. In our study, we also showed altered levels of arginine and ornithine, which corresponds to literature data [15]. Aspartate is a component of the malate shuttle, which is involved in energy production. The obtained reduced serum levels of aspartate in our study may imply deficits in neuronal activity and mitochondrial function in schizophrenia. Glycine functions as a coagonist along with glutamate or aspartate in the stimulation of glutamatergic *N*-methyl D-aspartate (NMDA) receptors [25]. The low levels of these amino acids may point to the hypofunction of NMDA receptor-mediated neurotransmission in the patients, which represents a key pathogenic mechanism of schizophrenia [26]. The spectrum of acylcarnitines includes a wide range of structures that is very different both chemically and metabolically. For instance, short-chain acylcarnitines are small, water-soluble molecules that are easily transportable to a variety of locations, whereas long-chain acylcarnitines need a transporter to cross the plasma membrane and, therefore, may be more restricted in their actions. As a result, changes in individual acylcarnitines may imply changes in specific metabolic pathways [8].

**Figure A1:**
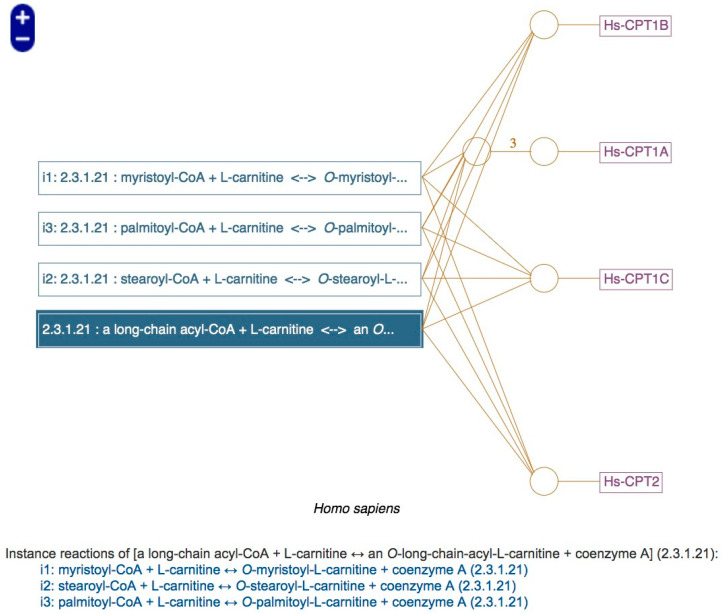
Metabolic pathway involving carnitine palmitoyltransferase I (CPT1) and O-long-chain-acyl-L-carnitine. The scheme is taken from the online resource metacyc.org with EC number 2.3.1.21. The Hs-CPT1C is associated with brain isoform of carnitine O-palmitoyltransferase 1.

The decreased level of long-chain acylcarnitines in schizophrenia, obtained in our study, may be caused by a decreased expression or activity of different transporters in the plasma membrane. Using the metabolic pathway analysis method on the metacyc.org online resource [27], we found that our findings are related to the activity of Carnitine palmitoyltransferase I (CPT1) (Figure A1). Changes in CPT activity have been observed in neurological and mental diseases, mainly associated with disturbances in the balance of insulin in the brain, such as Parkinson′s and Alzheimer′s diseases and schizophrenia [28]. In schizophrenia, B. Cao et al. showed a decrease both long- and medium-chain acylcarnitines [14]. However, in our study the levels of medium-chain acylcarnitines were comparable to the control group. Reduced levels of long-chain acylcarnitines after 7 months of antipsychotic drug treatment were shown in the study by K. Kriisa et al. [21]. However, antipsychotic-naïve first-episode psychosis patients demonstrated significantly increased levels of long-chain acylcarnitines in comparison to healthy controls [21]. Additionally, significantly lower levels of several medium- and long-chain acylcarnitines were shown in patients with diabetes mellitus (types 1 and 2) and metabolic syndrome [29] and usage of acylcarnitines as markers of metabolic syndrome was suggested [20]. Moreover, it was previously revealed that increased catabolism of branched-chain amino acid occurs and is correlated with insulin resistance and short-chain acylcarnitines [20,30]. Individuals with schizophrenia are known to have an increased risk of diabetes mellitus, metabolic syndrome, and other metabolic abnormalities [31]. In this case, schizophrenia itself may be a risk factor for metabolic syndrome, and also antipsychotic drugs, particularly second-generation antipsychotics, may contribute the risk [32]. Recently, it has been demonstrated that the level of acylcarnitines in patients with schizophrenia differs depending on the presence of metabolic syndrome [33]. 

As noted above all patients in our study were using antipsychotic therapy and had already been ill for a considerable number of years. In that regard, we do not indicate that it is the root cause of altered amino acid and acylcarnitine levels—schizophrenic process or metabolic side effects of antipsychotics. 

Limitations include the limited sample size, which was not characterized with respect to obesity, metabolic syndrome, diabetes mellitus, alcohol intake, and current drug treatment (particularly antipsychotics; but our patients did not take lipid-lowering or antidiabetics drugs). There is also the possibility that changes in amino acid and acylcarnitine levels can result from pharmacological intervention or diet. However, we tried to minimize these interventions by blood collection after a 12-h overnight fast. Given the factors, results should be interpreted with caution. The reported results provide rationale for the study of amino acid and acylcarnitine in larger sample cohorts patients with schizophrenia, in drug-naïve patients, and taking into account the effect of specific antipsychotics and diet habits.

## 4. Materials and Methods 

### 4.1. Participants

All subjects were inpatients from the Mental Health Research Institute Tomsk National Research Medical Center (Tomsk, Russia). The Local Bioethics Committee of the Mental Health Research Institute Tomsk National Research Medical Center (Tomsk, Russia) reviewed and approved the study protocols (#187, 24 April 2018). Each patient and healthy person provided written informed consent prior to their inclusion. All procedures were carried out in accordance with the Declaration of Helsinki 1975, revised in Fortaleza, Brazil, 2013. Each patient provided written informed consent after proper explanation. The inclusion criteria were a clinical diagnosis of schizophrenia, according to the International Statistical Classification of Diseases and Related Health Problems, 10th Revision (ICD-10: F20), and aged between 18 and 60 years old. The exclusion criteria were a history of acute and chronic infectious, autoimmune diseases, hereditary metabolic diseases, undergoing medical treatments with valproic acid, that can affect acylcarnitine levels.

The control group consisted of men and women, 18–60 years old with no schizophrenia as determined by the ICD-10 recruited on a voluntary basis.

### 4.2. Blood Sampling

All fasting blood samples (≈5 mL) were collected from subjects in the first days of hospitalization. Blood samples (≈5 mL) were drawn after a 12-h overnight fast in the first days of hospitalization before initiation of antipsychotic treatment into tubes with a clot activator (BD, Franklin Lakes, NJ, USA). Blood samples were centrifuged for 30 min at 2000× *g* at 4 °C to isolate the serum; the serum was stored at −80 °C, until analysis. The serum was plotted on the ProteinSaver card 903™ (Whatman^®^, Maidstone, UK) prior to analysis.

### 4.3. Amino Acid and Acylcarnitine Measurement

The serum spots were allowed to dry at room temperature for at least 3 h before analysis. Paper disks with a diameter of 3.2 mm were punched out with a hand puncher. Quantification of amino acids and acylcarnitines was carried out using as internal standard the isotope labeled standards from an Amino Acids and Acylcarnitines kit #55000 for newborn screening (Chromsystems Instruments and Chemicals, Munich, Germany). Sample preparation was done according to the kit manufacturer’s protocol from dried spots. Mass spectrometry analysis was carried out in the Core Facility of Mass Spectrometric Analysis of the Institute of Chemical Biology and Fundamental Medicine, Siberian Branch of the Russian Academy of Sciences. The amino acids and acylcarnitines were analyzed according to a standard procedure [34]. Each series of sample preparation included the QC quality control samples (L1 and L2 from kit #55000) with known concentrations of amino acids and acylcarnitines for control of sample preparation and mass spectrometric analysis.

Chromatographic separation of amino acids and acylcarnitines were performed on an Agilent 1210 LC system using a Prontosil 120-3-C18 AQ reversed-phase column (75 × 2.1 mm, 3 μm, Econova, Russia). The eluent A consisted of 2.5 mM solution of ammonium formate (pH 4), and eluent B was methanol containing 0.1% of formic acid. The flow rate was 0.15–0.25 mL/min within 20 min. Elution gradient was set as follows: 0–0.5 min: 1% B, 0.5–0.6 min: 1–30% B, 0.6–7.5 min: 30–95% B, 7.5–10 min: 95–100% B, 10–14 min: 100% B, 14–14.5 min: 100–1% B, 15–20 min: 1% B. The autosampler temperature was set at 4 °C, the injection volume was 5 µL, and the column was thermostated at 40 °C.

The mass spectrometric analysis was carried out in the dynamic multiple reaction monitoring (Dynamic MRM) mode using an Agilent 6410 QQQ mass spectrometer (Agilent Technologies, Palo Alto, CA, USA). Mass spectrometer parameters were set as follows: source temperature: 325 °C, gas flow: 8 L/min, nebulizer: 40 psi, capillary voltage: 4000 V, delta EMV 200 V, Q1 and Q3 resolution: unit. Other parameters, individual for each transition are presented in Supplementary Table S1.

Peak alignment, integration, and quantification were performed using MassHunter Quantitative Analysis software (Agilent Technologies, Palo Alto, CA, USA). The calculated concentrations of amino acids and acylcarnitines by the ratio of peak areas of analytes and internal standards were summarized in the final table in the Excel file format for subsequent data analysis.

### 4.4. Statistical Analysis

Statistical analysis and visualization were carried out in the scripting language R v. 3.6.1 with RStudio environment v. 1.2.5001 using the packages “dplyr”, “caret”, “factoextra”, “gglplot2” and others. Differences in general characteristics between the studied groups were tested using *χ*^2^ tests, Student′s *t*-tests, or Mann-Whitney U-tests. Differences in levels of amino acid and acylcarnitine between the groups were tested using Mann-Whitney U-tests. Multivariate analyses were included the hierarchical cluster analysis and the PLS-DA. *p*-values less than 0.05 were regarded as statistically significant.

## 5. Conclusions

In summary, patients with schizophrenia had an altered concentration of a few amino acids and acylcarnitines in comparison to the healthy probands. Further research is needed to assess and understand the identified changes.

## Figures and Tables

**Table 1:** The general characteristic of patients with schizophrenia and healthy probands.

Indicators	Patients with Schizophrenia (*n* = 37)	Healthy Probands (*n* = 36)	*p*-Value
Age, Me (Q1; Q3), years	35 (31.00; 39.00)	32.5 (28.75; 40.25)	0.798
Gender (male, *n* (%)/female, *n* (%))	19 (51.3)/18 (48.7)	22 (61.1)/14 (38.9)	0.340
Duration of disease, mean ± SD, years	15 ± 8.5	N/A	N/A
BMI	24.1 (22.3; 25.9)	22.6 (21.9; 25.0)	0.511

Me (Q1; Q3), median (lower quartile; upper quartile); N/A—not applicable; BMI—body mass index.

**Table 2:** The concentration of amino acids (μmol/L) in the blood serum of patients with schizophrenia and healthy individuals, Me (Q1;Q3).

Amino Acid Level	Patients with Schizophrenia (*n* = 37)	Healthy Individuals (*n* = 36)	*p*-Value
Alanine	152.16 (135.18; 196.56)	158.69 (141.60; 187.45)	0.631
Arginine	48.538 (42.50; 54.13)	56.16 (50.42; 62.44)	0.002 *
Aspartate	21.07 (17.71; 24.82)	29.67 (26.74; 36.58)	<0.001 *
Citrulline	18.94 (16.71; 21.75)	22.65 (19.87; 26.73)	<0.001 *
Glycine	126.39 (106.77; 144.76)	148.32 (126.64; 169.50)	0.003 *
Methionine	14.76 (10.68; 16.53)	15.71 (13.40; 17.71)	0.210
Ornithine	83.64 (76.87; 94.18)	95.06 (81.12; 108.42)	0.021 *
Phenylalanine	28.47 (26.06; 37.49)	32.05 (29.71; 33.68)	0.482
Tyrosine	29.28 (23.81; 34.86)	32.55 (27.49; 38.35)	0.097
Valine	89.80 (78.42; 107.06)	109.26 (93.49; 121.08)	<0.001 *
Leucine/isoleucine	56.15 (48.03; 68.76)	57.27 (50.53; 69.55)	0.858
Proline	99.70 (82.22; 123.27)	92.20 (77.65; 114.56)	0.172
Alanine	75.40 (64.82; 94.60)	158.69 (141.60; 187.45)	0.631
Arginine	126.39 (106.77; 144.76)	56.16 (50.42; 62.44)	0.002 *
Aspartate	14.76 (10.68; 16.53)	29.67 (26.74; 36.58)	<0.001 *

Me (Q1; Q3), median (lower quartile; upper quartile); * *p* < 0.05, statistically significant difference.

**Table 3:** The concentration of acylcarnitine (µmol/L) in the blood serum of patients with schizophrenia and healthy individuals, Me (Q1; Q3).

Acylcarnitine Level	Patients with Schizophrenia (*n* = 37)	Healthy Individuals (*n* = 36)	*p*-Value
C0	13.6498 (12.4162; 16.8279)	14.5119 (11.8942; 18.3337)	0.547
C2	17.7022 (12.9025; 21.0256)	17.5045 (13.7932; 19.8577)	0.872
C3	0.4098 (0.3502; 0.5662)	0.3949 (0.3127; 0.4762)	0.156
C3-DC	0.0906 (0.0772; 0.1069)	0.0917 (0.0738; 0.1062)	0.795
C4	0.0765 (0.0646; 0.1115)	0.0790 (0.0608; 0.0896)	0.443
C4-OH	0.0095 (0.0078; 0.0134)	0.0104 (0.0079; 0.0138)	0.855
C4-DC	0.0327 (0.0286; 0.0365)	0.0248 (0.0220; 0.0279)	<0.001 *
C5	0.0389 (0.0320; 0.0490)	0.0394 (0.0324; 0.0472)	0.574
C5-OH	0.0227 (0.0204; 0.0253)	0.0224 (0.0203; 0.0254)	0.511
C5:1	0.0061 (0.0053; 0.0076)	0.0078 (0.0069; 0.0099)	<0.001 *
C5-DC	0.0698 (0.0585; 0.0869)	0.0792 (0.0547; 0.1026)	0.312
C6	0.0224 (0.0196; 0.0319)	0.0299 (0.0225; 0.0393)	0.099
C8	0.0413 (0.0270; 0.0611)	0.0504 (0.0336; 0.0767)	0.187
C8:1	0.0279 (0.0203; 0.0349)	0.0246 (0.0166; 0.0339)	0.126
C10	0.0532 (0.0351; 0.0755)	0.0576 (0.0356; 0.0834)	0.615
C10:1	0.0671 (0.0464; 0.1033)	0.0566 (0.0454; 0.0790)	0.169
C12	0.0200 (0.0127; 0.0254)	0.0229 (0.0169; 0.0331)	0.080
C14	0.0097 (0.0085; 0.0122)	0.0121 (0.0107; 0.0149)	0.019*
C14-OH	0.0023 (0.0019; 0.0030)	0.0030 (0.0025; 0.0039)	0.018 *
C14:1	0.0172 (0.0130; 0.0267)	0.0180 (0.0140; 0.0267)	0.719
C14:2	0.0130 (0.0081; 0.0187)	0.0129 (0.0085; 0.0177)	0.855
C16	0.0291 (0.0238; 0.0335)	0.0332 (0.0251; 0.0356)	0.155
C16-OH	0.0018 (0.0015; 0.0025)	0.0038 (0.0031; 0.0045)	<0.001 *
C16:1	0.0067 (0.0049; 0.0097)	0.0097 (0.0084; 0.0120)	0.003 *
C16:1-OH	0.0027 (0.0020; 0.0032)	0.0060 (0.0049; 0.0068)	<0.001 *
C18	0.0125 (0.0105; 0.0157)	0.0248 (0.0215; 0.0289)	<0.001 *
C18-OH	0.0031 (0.0025; 0.0046)	0.0051 (0.0044; 0.0061)	<0.001 *
C18:1	0.0297 (0.0263; 0.0359)	0.0348 (0.0304; 0.0416)	0.029 *
C18:1-OH	0.0023 (0.0019; 0.0030)	0.0069 (0.0054; 0.0086)	<0.001 *
C18:2-OH	0.0028 (0.0021; 0.0040)	0.0038 (0.0031; 0.0044)	0.003 *

Me (Q1; Q3), median (lower quartile; upper quartile); * *p* < 0.05, statistically significant difference.

## Data Availability

Data available on request due to privacy and ethical restrictions.

## Supplementary Materials

The following are available online at https://www.mdpi.com/2218-1989/11/1/34/s1, Table S1: The details of dynamic MRM parameters.
